# Timing of Community Mitigation and Changes in Reported COVID-19 and Community Mobility ― Four U.S. Metropolitan Areas, February 26–April 1, 2020

**DOI:** 10.15585/mmwr.mm6915e2

**Published:** 2020-04-17

**Authors:** Arielle Lasry, Daniel Kidder, Marisa Hast, Jason Poovey, Gregory Sunshine, Kathryn Winglee, Nicole Zviedrite, Faruque Ahmed, Kathleen A. Ethier, CDC Public Health Law Program, New York City Department of Health and Mental Hygiene, Louisiana Department of Health, Public Health – Seattle & King County, San Francisco COVID-19 Response Team, Alameda County Public Health Department, San Mateo County Health Department, Marin County Division of Public Health, CDC Public Health Law Program, Catherine Clodfelter, Mara Howard-Williams, Rachel Hulkower, Gi Jeong, Lisa Landsman, Russell McCord, Amanda Moreland, Julia Shelburne, Louisiana Department of Health, Alexander Billioux, Julie Hand, Joseph Kanter, Andrew Smith, Theresa Sokol, Public Health – Seattle & King County, Jeffrey S. Duchin, Meaghan S. Fagalde, Sargis Pogosjans, Alameda County Public Health Department, Robert Brown, Sandra Huang, Nicholas Moss, Erica Pan, Munira Shemsu, Emily Yette, San Mateo County Health Department, Carly Bock, Rachel Curtis-Robles, Cassius Lockett, Scott Morrow, Catherine Sallenave, Marin County Division of Public Health, Lisa Santora, Matthew Willis

**Affiliations:** ^1^CDC COVID-19 Response Team; ^2^Epidemic Intelligence Service, CDC; ^3^Georgia Tech Research Institute, Atlanta, Georgia; ^4^Public Health Law Program, CDC.; CDC Public Health Law Program; CDC Public Health Law Program; CDC Public Health Law Program; CDC Public Health Law Program; CDC Public Health Law Program; CDC Public Health Law Program; CDC Public Health Law Program; CDC Public Health Law Program.; Louisiana Department of Health; Louisiana Department of Health; Louisiana Department of Health; Louisiana Department of Health; Louisiana Department of Health.; Public Health – Seattle & King County; Public Health – Seattle & King County; Public Health – Seattle & King County.; Alameda County Public Health Department; Alameda County Public Health Department; Alameda County Public Health Department; Alameda County Public Health Department; Alameda County Public Health Department; Alameda County Public Health Department.; San Mateo County Health Department; San Mateo County Health Department; San Mateo County Health Department; San Mateo County Health Department; San Mateo County Health Department.; Marin County Division of Public Health; Marin County Division of Public Health.

Community mitigation activities (also referred to as nonpharmaceutical interventions) are actions that persons and communities can take to slow the spread of infectious diseases. Mitigation strategies include personal protective measures (e.g., handwashing, cough etiquette, and face coverings) that persons can use at home or while in community settings; social distancing (e.g., maintaining physical distance between persons in community settings and staying at home); and environmental surface cleaning at home and in community settings, such as schools or workplaces. Actions such as social distancing are especially critical when medical countermeasures such as vaccines or therapeutics are not available. Although voluntary adoption of social distancing by the public and community organizations is possible, public policy can enhance implementation. The CDC Community Mitigation Framework (*1*) recommends a phased approach to implementation at the community level, as evidence of community spread of disease increases or begins to decrease and according to severity. This report presents initial data from the metropolitan areas of San Francisco, California; Seattle, Washington; New Orleans, Louisiana; and New York City, New York* to describe the relationship between timing of public policy measures, community mobility (a proxy measure for social distancing), and temporal trends in reported coronavirus disease 2019 (COVID-19) cases. Community mobility in all four locations declined from February 26, 2020 to April 1, 2020, decreasing with each policy issued and as case counts increased. This report suggests that public policy measures are an important tool to support social distancing and provides some very early indications that these measures might help slow the spread of COVID-19.

When a novel virus with pandemic potential emerges, community mitigation strategies often are the most readily available interventions to slow transmission. CDC-recommended community mitigation interventions for COVID-19, caused by the SARS-CoV-2 virus, are based on evidence for other viral respiratory illnesses and emerging data on SARS-CoV-2 transmission and epidemiology, including groups at highest risk for hospitalization and death from COVID-19 (*1*,*2*).

Public policies to implement social distancing include emergency declarations, bans on gatherings of certain sizes, school closures, restrictions on businesses, and stay-at-home or shelter-in-place of residence orders. These strategies can substantially disrupt daily life; therefore, the intensity of their implementation should align with progression and severity of disease (*1*). Understanding the timing and potential impact of policies designed to increase compliance with mitigation strategies will assist in guiding modification of those policies over the course of the COVID-19 pandemic as well as increasing the understanding of when and how to fully implement these strategies in future outbreaks where community mitigation is required.

Data from February 26–April 1, 2020 were examined from the core metropolitan statistical areas (MSAs) of Seattle, San Francisco, and New Orleans, and from the five boroughs of New York City (*3*). These areas were selected because each had substantial numbers of reported COVID-19 cases during the early stages of the U.S. epidemic (*4*). For each locality, the following data were analyzed: 1) types and timing of public policies issued to promote community mitigation interventions at the national, state, and local government levels; 2) cumulative number of reported COVID-19 cases; 3) average 3-day percentage change in reported cases; and 4) community mobility.

The types and timing of public policies issued were collected by using Google Alerts and targeted Google searches for news media coverage of state and local COVID-19 orders and proclamations, followed by searching state, county, parish, and city government websites to locate official copies of each order. Confirmed cumulative COVID-19 case count data were collected from USAFacts (*4*), which aggregates data on cases by date of report from CDC and state- and local-level public health agencies. The 3-day average percentage change in cumulative case count was calculated after the cumulative case count was >20 and is presented to describe more completely the trend in the epidemic growth rate. Community mobility was defined as the percentage of personal mobile devices (e.g., mobile phones, tablets, and watches) leaving home, using publicly accessible data from SafeGraph, a data company that aggregates anonymized location data from mobile devices (*5*). The percentage leaving home measure is the inverse of the SafeGraph “completely home” metric, an indicator that a device has not moved throughout the day beyond approximately 150 m (492 ft) of its common nighttime location. The average number of devices included in daily reporting was 80,095 in New Orleans (6.4% of population); 336,783 devices in New York City (4.0% of population); 163,981 devices in San Francisco (3.6% of population); and 177,027 devices in Seattle (4.8% of population).

In each of the four locations, a combination of state and local community mitigation policies was issued (Table). All four metropolitan areas were in states that declared a state of emergency and put local limits on mass gatherings, although these varied by numbers of people allowed and, in some cases, changed over time. All four issued school closure and stay-at-home orders at state or local levels, and three parishes in the New Orleans MSA were the only areas in this study to implement a curfew.

**TABLE:** Public policies ordering COVID-19 community mitigation interventions and dates of issuance* — four U.S. metropolitan areas, February 26–April 1, 2020

Mandatory intervention	New Orleans MSA parishes: Jefferson, Orleans, Plaquemines, St. Bernard, St. Charles, St. James, St. John the Baptist, St. Tammany	New York City boroughs: The Bronx, Brooklyn, Manhattan, Queens, Staten Island	San Francisco MSA counties: Alameda, Contra Costa, San Francisco, San Mateo, Marin	Seattle MSA counties: King, Snohomish, Pierce
State declaration of emergency	March 11	March 7	March 4	February 29
Local declaration of emergency	March 11: Orleans	March 12: New York City	February 25: San Francisco	March 2: King
	March 12: Jefferson		March 1: Alameda	March 3: City of Seattle
	March 13: St. Tammany, St. James		March 3: San Mateo, Marin	March 4: Snohomish
	March 14: St. Charles		March 10: Contra Costa	
	March 15: Plaquemines, St. John the Baptist			
	March 16: St. Bernard			
State limits on mass gatherings	March 13: limiting to <250	March 12: limiting to <500	March 11: limiting to <250	March 11: limiting to <250 for King, Pierce, Snohomish
	March 16: limiting to <10	March 16: limiting to <50		March 15: limiting to <50 statewide
		March 23: banning all non-essential gatherings		
Local limits on mass gatherings^†^	March 16: City of New Orleans, Orleans Parish canceling all public gatherings	March 15: New York City limiting to <500	March 11: San Francisco limiting to <1,000	March 11: Public Health – Seattle & King County limiting to <250
		March 20: New York City limiting to <50	March 12: San Mateo – limiting to <250	
		March 25: New York City banning all non-essential gatherings	March 13: San Francisco limiting to <100	
			March 14: Contra Costa limiting to <100; San Mateo limiting to <50	
State limits on senior living facilities	March 12^§^	March 12	NA^¶^	March 10: limiting visitors
				March 16: banning visitors
Local limits on senior living facilities	NR	NR	March 11: San Mateo	NR
			March 12: San Francisco	
State school closure	March 13	March 16	NA**	March 12: state order for King, Pierce, Snohomish
				March 13: statewide
Local school closure	NR	March 15: New York City	March 13: Marin, San Mateo^††^	NA^§§^
State limits on bars and restaurants	March 16	March 16	March 19	March 16
Local limits on bars and restaurants	March 16: Orleans, City of New Orleans	March 16: New York City	NR	NR
State stay-at-home/shelter-in-place order	March 22	March 20	March 19	March 23
Local stay-at-home/shelter-in-place order	March 20: Orleans, City of New Orleans	March 20: New York City	March 16: Alameda, Contra Costa, Marin, San Francisco, San Mateo	March 24: Snohomish
Local curfew order	April 1: St. James, St. John the Baptist	NR	NR	NR
	April 2: Plaquemines			

**Abbreviations**: COVID-19 = coronavirus disease 2019; MSA = metropolitan statistical area; NA = not applicable; NR = none reported.

* Issuance dates are the dates the issuing official signed the order implementing the mandatory intervention. In some instances, interventions were effective either immediately or within 1–3 days of issuance. Recommendations and guidance are not included.

^†^ Dates reflect issuance of mandated restrictions on the size of mass gatherings. The cancellation of individual events is excluded.

^§^ Visitor limitations were issued for all licensed health care providers.

^¶^ Guidance states that the March 19 stay-at-home order “prohibits non-necessary visitation to these kinds of facilities except at the end-of-life.”

** Although no statewide school closure mandate was issued, the governor’s March 13 Executive Order N-26–20 describes multiple orders applicable to local educational agencies that choose to close to address COVID-19.

^††^ Schools also closed in other San Francisco MSA counties, but decisions were made at the school district level and not as mandatory policies implemented at the municipal, county, or state level.

^§§^ Schools districts in the Seattle MSA began closing on March 11, but these decisions were made at the district level and not as mandatory policies implemented at the municipal, county, or state level.

In addition to state and local policies, which were implemented beginning in March, on March 16, 2020, the White House announced the 15 Days to Slow the Spread guidelines for persons to take action to reduce the spread of COVID-19. This national action was extended for an additional 30 days on March 30, 2020.^†^

Timing of community mitigation policies in relation to the increasing cumulative case counts of COVID-19 varied by locality (Figure). In all four metropolitan areas, an emergency declaration was the first policy issued, before large increases in cumulative cases. Stay-at-home orders were the last mitigation policy to be issued in all areas except for the New Orleans MSA, where a curfew in three of eight parishes was issued after the stay-at-home order. In all four metropolitan areas, the percentage of residents leaving home declined as the number of policies issued increased (Figure); in all four localities the percentage leaving home was close to 80% on February 26, and by April 1 the percentage leaving home was 42% in New York City, 47% in San Francisco, 52% in Seattle, and 61% in New Orleans. Overall, across the four areas, emergency declarations (the first policies issued) did not result in a sustained change in mobility; however, declines in mobility occurred after implementation of combinations of policies (such as limits on gatherings or school closures) and after the White House 15 Days to Slow the Spread guidelines were implemented. There were additional declines in mobility following stay-at-home orders in all four locations. The average 3-day percentage change also varied by locality, with some variation across the four metropolitan areas during the first two weeks of March, followed by a decline and leveling in the last two weeks of March. These changes also follow the issuance of a set of policies and rapid decline in mobility mid-March. 

**FIGURE:**
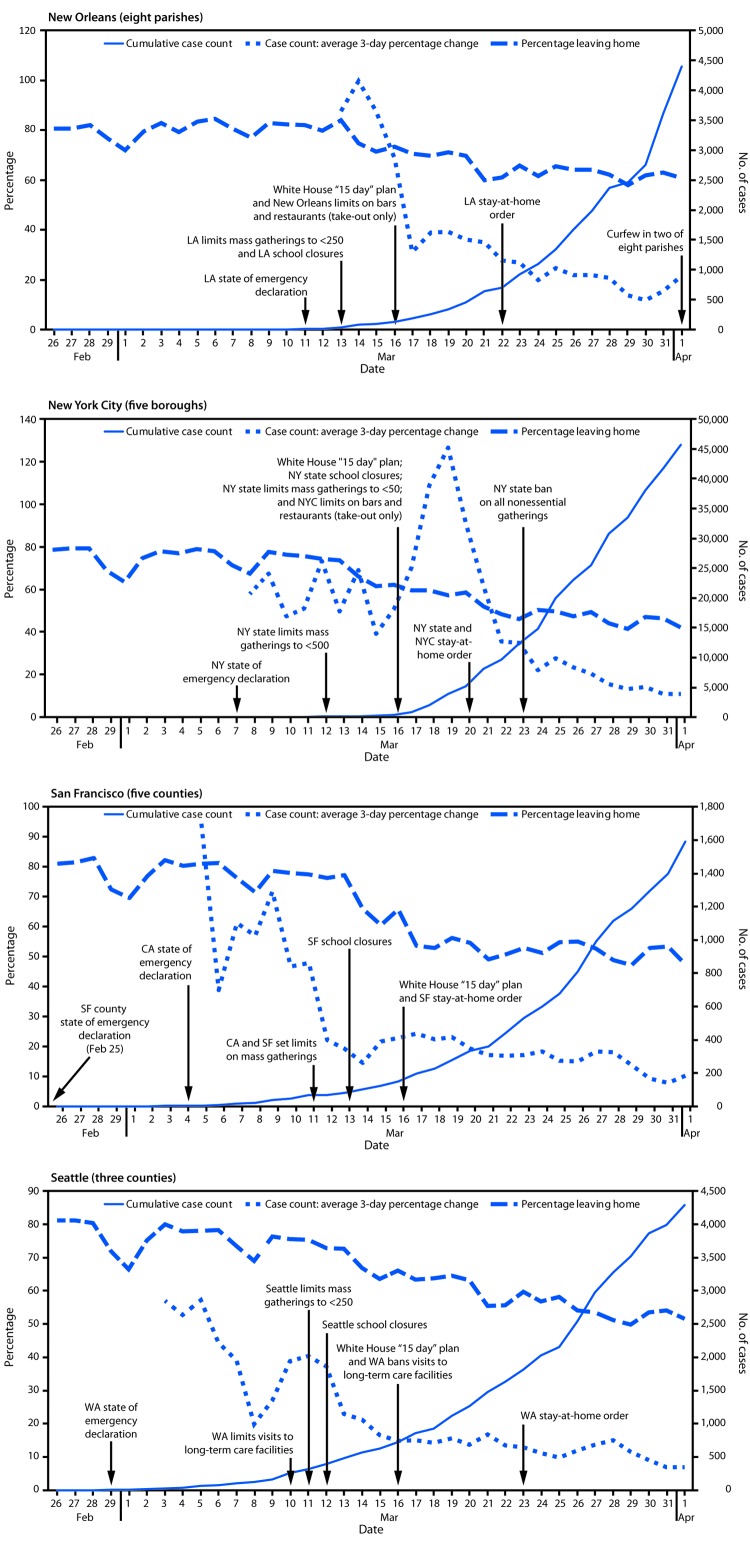
Selected community mitigation interventions,* cumulative COVID-19 case counts, average 3-day percentage change in case counts,**^†^** and percentage leaving home — four U.S. metropolitan areas,^§,¶^ February 26–April 1, 2020 **Abbreviations:** CA = California; COVID-19 = coronavirus disease 2019; LA = Louisiana; NY = New York; NYC = New York City; SF = San Francisco; WA = Washington. * Public policies ordering COVID-19 community mitigation interventions presented by date of issuance. ^†^ Plotting of average 3-day percentage change begins when cumulative case count >20. ^§^ San Francisco metropolitan statistical area (MSA) counties include Alameda, Contra Costa, San Francisco, San Mateo, and Marin; Seattle MSA counties include King, Snohomish, and Pierce; New York City boroughs include The Bronx, Brooklyn, Manhattan, Queens, and Staten Island; New Orleans MSA parishes include Jefferson, Orleans, Plaquemines, St. Bernard, St. Charles, St. James, St. John the Baptist, and St. Tammany. ^¶^ The primary and secondary vertical axis are different across locations and set according to each location’s data.

## Discussion

During February 26–April 1, 2020, as cumulative cases increased and community mitigation policies were implemented, community mobility declined in four U.S. metropolitan areas. With the exception of emergency declarations, which were implemented as cases increased in other regions and internationally, these policies were implemented during the period when case counts were increasing in each location, but the timing in relation to cumulative case counts varied. Public policies to increase compliance with social distancing, including limits on mass gatherings, school closures, business restrictions, and stay-at-home or shelter-in-place orders appear to be associated with decreases in mobility. Policies related to specific locations or community organizations (e.g., mass gatherings, schools, restaurants, and bars) were often implemented within one or two weeks of mid-March, likely a result of increased awareness and concern about the potential scope of the outbreak in the absence of mitigation. This awareness and concern also likely impacted the public, potentially leading to further decreases in mobility. Thus, the potential impact of interventions on mobility as well as this increased awareness of community spread of disease appears to be cumulative over time. Monitoring adherence to community mitigation strategies through mobility measures could improve the understanding of the types, combinations, and timing of policies that are associated with slowing the spread of COVID-19 as well as other infectious diseases. Finally, there appears to be very early indications of potential impact of policies and social distancing on later changes in cases. There are likely a variety of contributors to these changes, including public health efforts to contain spread and individual efforts to increase personal protective practices. However, both policies related to community mitigation and social distancing, operationalized here as community mobility, could have contributed to these changes.

The findings in this report are subject to at least four limitations. First, these data suggest temporal correlations between issuance of public policies to increase mitigation strategies and rising case counts, on one hand, and decreases in mobility, on the other as well as first indications that these changes might impact growth of infections. The trends suggest an association but cannot prove causality. Second, although mobile device data can be used to understand movement within a community, the characteristics of those persons using these devices (e.g., age, gender, race, and ethnicity) are not known, so the results might not be generalizable or reflective of actual mobility patterns. Further, mobile phone coverage was limited to 3%–6% of the population in each location. In addition, the data presented here track mobile devices, not persons, who might have multiple devices (e.g., phone and tablet), who might not take their devices when they leave the home, or who might travel outside their home but remain within 150 m (492 ft) of their usual nighttime location. Third, confirmed cumulative cases of COVID-19 might not reflect the actual number of cases because of variability in access to testing and recommendations for who should be tested during this period. Finally, these four urban metropolitan areas are not representative of communities across the United States, and community mitigation policies might have a very different impact on mobility in suburban and rural communities.

These temporal trend data provide a preliminary examination of local timing of community mitigation measures and potential impacts on community mobility as well as very early indications of the impact of community mitigation on disease growth. As the COVID-19 pandemic spreads across the United States, the ability to assess the impact of mitigation strategies on reducing COVID-19 transmission will improve. Decreasing numbers of new cases are needed to curtail the COVID-19 pandemic in communities and relieve pressure on the health care system. Better understanding of the short- and long-term impact of the community disruption that results from these measures is critical. However, this analysis suggests that policies to increase social distancing when case counts are increasing can be an important tool for communities as changes in behavior result in decreased spread of COVID-19.

**Summary**

**What is already known on this topic?**

Implementing community mitigation strategies, including personal protective measures persons should adopt in community settings, social distancing, and environmental cleaning in community settings, during a pandemic can slow the spread of infections.

**What is added by this report?**

During February 26–April 1, 2020, community mobility (a proxy measure for social distancing) in the metropolitan areas of Seattle, San Francisco, New York City, and New Orleans declined, decreasing with each community mitigation policy issued and as case counts increased.

**What are the implications for public health practice?**

Public policies to increase compliance with community mitigation strategies might be effective in decreasing community mobility; however, more information is needed to assess impact on disease transmission.

## Footnotes

* San Francisco metropolitan statistical area (MSA) counties include Alameda, Contra Costa, San Francisco, San Mateo, and Marin; Seattle MSA counties include King, Snohomish, and Pierce; New York City boroughs include The Bronx, Brooklyn, Manhattan, Queens, and Staten Island; New Orleans MSA parishes include Jefferson, Orleans, Plaquemines, St. Bernard, St. Charles, St. James, St. John the Baptist, and St. Tammany.

† https://www.whitehouse.gov/wp-content/uploads/2020/03/03.16.20_coronavirus-guidance_8.5x11_315PM.pdf.
